# Determination of Normal Anal Position Using the Anal Position Index in Pakistani Neonates

**DOI:** 10.7759/cureus.45144

**Published:** 2023-09-13

**Authors:** Areej Salim, Muhammad Awais Kanwal, Zainab Haji, Kashaf Turk, Yusra Tariq, Umaisa Khalid, Muhammad Arif Mateen Khan

**Affiliations:** 1 Pediatrics Surgery, Aga Khan University Hospital, Karachi, PAK; 2 Surgical Oncology, Shaukat Khanum Memorial Cancer Hospital and Research Centre, Lahore, PAK; 3 Dental Surgery, Aga Khan University Hospital, Karachi, PAK

**Keywords:** pediatric surgery, ada, anterior displacement of anus, api, normal anal position, anal position index, pakistani neonates

## Abstract

Introduction: Anterior displacement of the anus (ADA) is recognized as a common congenital abnormality of the anorectal region and is often associated with constipation. It is diagnosed through a physical examination by measuring the Anal Position Index (API) at birth.

Methods: A cross-sectional study was conducted utilizing non-probability consecutive sampling of all patients presenting with ADA at our institute over a six-month period. The study focused on key variables, including the measurement of scroto-anal and scroto-coccygeal distances in boys and fourchette-anal and fourchette-coccygeal distances in girls. Data collection was carried out using a structured proforma, ensuring prospective data collection from neonates meeting the selection criteria (neonates born at our center and identified by the neonatology team as having a normal anal opening, regardless of their gestational period or birthweight).

Results: Our study comprised a cohort of 204 neonates. The Anal Position Index was determined to be 0.36 ± 0.07 for male newborns and 0.24 ± 0.06 for female newborns. Notably, a statistically significant correlation was observed between the API and factors such as gestational age, birth weight, and advancing paternal age.

Conclusion: The assessment of the API proves highly valuable in identifying indicators of ADA in neonates, facilitating early disease detection, and guiding the prompt management of subsequent functional symptoms, such as constipation, during later stages of life.

## Introduction

Constipation is one of the most common disorders of anorectal function in childhood and infancy. Functional constipation is the most common cause of constipation; however, anatomical malformations in the anorectal area may be accountable in some cases [1,2]. There is a wide spectrum of severity for congenital anorectal anomalies. Anteriorly located anus or short perineal body, anterior anus, anteriorly displaced anus, and anterior ectopic anus are synonyms for the same malformation. It is thought to be due to the weakness of a segment of the anal canal with a corresponding malformation of the mid-portion of the external sphincter [3].

Anterior displacement of the anus (ADA) is reported in the literature as a common developmental anomaly and is the least severe form of anorectal malformation [4].

ADA stands as a frequently encountered congenital anomaly of the anorectum, predominantly manifesting as constipation in infants and children [5]. The diagnosis of ADA traditionally relies on a visual inspection of the anus. However, contemporary scientific methodologies have introduced a range of tools for quantifying the normal positioning of the anus in neonates. Foremost among these innovations is the pioneering work of Reisner et al. [6]. Their proposed "Anal Position Index" (API) offers a quantitative approach to determining the position of the anal opening on the perineum, thereby establishing objective diagnostic criteria.

The API employs specific formulas for its calculation, distinct for boys and girls, as follows:

Anal Index (boys): Scroto-anal distance (cm)/Scroto-coccygeal distance (cm)

Anal Index (girls): Fourchette-anal distance (cm)/Fourchette-coccygeal distance (cm)

According to the research team, an anal index of less than 0.34 in girls and less than 0.46 in boys signifies the presence of this pathological condition, with a higher prevalence observed among girls [5].

ADA frequently coexists with developmental abnormalities in the mid-part of the external anal sphincter, leading to weakened functioning of the corresponding segment of the anal canal. This condition typically presents either at birth or during the post-weaning period and is characterized by constipation and discomfort during defecation. In cases where medical interventions fail to alleviate constipation, surgical correction of ADA has emerged as the definitive therapeutic approach. Surgical management often results in the formation of a prominent posterior rectal shelf (PRS) and a posterior anal dimple located behind the anus [7].

While many countries have adopted the formula proposed by Reisner et al. to compute the API for infants within their populations, there is a dearth of literature addressing this matter within the Pakistani community. Consequently, our study serves as the primary dataset from Pakistan, contributing essential insights to this scientific domain.

The objective of this study was *to assess and determine the normal anal position in Pakistani neonates using the Anal Position Index suggested by Reisner et al. at a tertiary care university hospital*.

## Materials and methods

Study design

This observational study was meticulously executed within the Department of Pediatric Surgery at our esteemed institute. Employing a systematic non-probability consecutive sampling approach, we aimed to comprehensively evaluate and ascertain the normal anal position in Pakistani neonates presenting with anorectal developmental anomalies (ADA).

Sample size determination

Our sample size determination was anchored in a meticulous analysis of prior research employing the sophisticated PASS 2011 software. Building upon the findings of Mohta et al. [8], who reported the mean normal anal position as 0.43 (SD=0.05) for males and 0.37 (SD=0.06) for females, we endeavored to derive an accurate estimate of the mean normal anal position within the range of 0.05 to 0.06. This endeavor led us to ascertain that a sample size of 204 participants would yield a two-sided 99% confidence interval with a margin of error equal to 0.02 (distance from the mean = 2.58 × 0.06/Sqrt(100) = 0.015, approx. = 0.02); 102 males and 102 females were thoughtfully enrolled.

Patient selection

The judicious selection of participants formed the cornerstone of our study. Inclusive criteria encompassed all neonates, regardless of gestational age or birth weight, born at our center and adjudged to possess a normal anal opening by the neonatology team. In a concerted effort to maintain precision and integrity in our investigation, exclusion criteria were meticulously crafted. Neonates presenting with ambiguous genitalia, coexisting anomalies of the lumbosacral region, cloacal anomalies, anterior ectopic anus, or imperforate anus with or without perineal fistulas were thoughtfully excluded.

Data collection

Adhering to the highest ethical standards, this study obtained the requisite ethical approval from the Ethics and Review Committee of our esteemed institution, marked by reference ID: 2020-3352-7242. The systematic and meticulous collection of data unfolded through the scrupulous use of a specially designed, structured proforma. This proforma served as the conduit through which a wealth of pertinent information was meticulously acquired from neonates meeting the meticulously defined selection criteria. Table 1 shows the data collection Proforma.

**Table 1:** Data collection Proforma.

Serial Number			
Gender	( ) Male ( ) Female		
Gestational age (weeks)			
Height (cm) at birth			
Weight (kg) at birth			
Head circumference (cm)			
Maternal age (years)			
Paternal age (years)			
Is the marriage consanguineous? (first degree)	( ) Yes ( ) No		
Maternal co-morbid (s)	( ) Yes ( ) No IF Yes (please choose at least one or more) ( ) Diabetes ( ) Gestational diabetes ( ) Hypertension ( ) Pregnancy induced hypertension ( ) Preeclampsia ( ) Eclampsia ( ) Hypothyroidism ( ) Hyperthyroidism		
Type of delivery	( ) Emergency ( ) Elective ( ) Spontaneous vaginal Delivery ( ) Assisted ( ) C-Section		
Associated congenital heart defect in the neonate	( ) Yes ( ) No		
Meconium passed (hours after birth)			
MALE BABY	Anus to scrotum distance (cm)	Coccyx to scrotum distance (cm)	API
	FEMALE BABY	Anus to fourchette distance (cm)	Coccyx to fourchette distance (cm)	

Data analysis

The intricate fabric of our data was meticulously analyzed through the sophisticated IBM SPSS Version 21 software (IBM Corp., Armonk, NY). A comprehensive presentation of patient demographics was meticulously crafted, skillfully employing frequencies and percentages to elucidate the pertinent aspects of our participant pool. The anthropometric measurements, spanning height, weight, and fronto-occipital circumference, were rendered tangible through the calculation of means and standard deviations. A quintessential component of our analysis was the computation of the API as a continuous variable. To glean deeper insights into the determinants of API values, a robust multivariate regression analysis was meticulously employed. This analysis specifically scrutinized the role of pivotal neonatal factors, including gender, gestational age, and consanguinity, in influencing the calculated API values.

Ethical considerations

Ethical considerations occupied a paramount position throughout the course of this investigation. The cardinal principles of confidentiality and anonymity were upheld with unwavering commitment. Sensitive data pertaining to participants was shrouded in a veil of utmost secrecy, as each data collection form was adorned with distinctive study-specific serial numbers. To fortify the safeguarding of sensitive medical information, a meticulously constructed password-protected file was meticulously established. This confidential repository safeguarded the linkage between medical record numbers and the aforementioned serial numbers, a realm exclusively accessible to the discerning eyes of the primary investigator.

## Results

A total of 204 neonates were included in our study. An even mix of both sexes (102 each) formed the cohort. In terms of birth weight, approximately 143 (70.1%) of the individuals had normal birth weight (>2.50 kg), 10 (4.9%) neonates had low birth weight (1.5 < n < 1 kg), and 7 (3.4%) newborns were labelled as having very low birth weight (<1 kg). About 125 (61.3%) of the neonates were born at term (more than 37 weeks of gestation). The mean height of the neonates was calculated at 48.78 ± 4.85 cm, birth weight at 2.62 ± 0.69 kg, and FOC at 33.23 ± 2.94 cm. Detailed patient characteristics can be better visualized in Table 2.

**Table 2:** Detailed patient characteristics.

Characteristics	n	%
Gender		
Female	102	50.0
Male	102	50.0
Birth weight (kg)		
≥2.5	143	70.1
<2.5 to ≥1.5	44	21.6
≤1.5 to ≥1.0	10	4.9
<1 kg	7	3.4
	Mean	S.D.
Height (cm)		
Female	48.53	4.799
Male	49.02	4.923
FOC (cm)		
Female	33.08	2.821
Male	33.37	3.078
	n	%
Gestational age in weeks		
≥37 to <42weeks	125	61.3
>32 to <37	56	27.5
≥28 to ≤32	19	9.3
<28	4	2.0
Consanguinity		
Yes	19	9.3
No	185	90.7
Maternal co-morbidities		
None	144	70.6
Diabetes	0	0.0
Gestational diabetes	27	13.2
Hypertension	0	0.0
Pregnancy-induced hypertension	16	7.8
Preeclampsia	1	0.5
Eclampsia	0	0.0
Hypothyroidism	10	4.9
Hyperthyroidism	1	0.5
PIH and GDM	5	2.5
	Mean	S.D.
Maternal age (years)		
Female	29.32	4.733
Male	29.39	4.560
Paternal age (years)		
Female	33.53	4.996
Male	33.76	5.682

**Figure 1:**
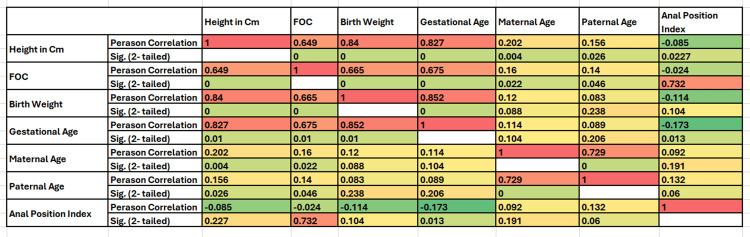
Detailed subgroup analysis.

The average value of API as suggested by the abovementioned formula was calculated at 0.30 ± 0.09 - boys: 0.36 ± 0.07 (range: 0.14 to 0.54); girls: 0.24 ± 0.06 (range: 0.13 to 0.49). The calculated value of API was significantly different in both genders at p< 0.001. Subgroup analysis showed that the API was significantly (p = 0.002) influenced by gestational age, with the majority of the difference observed between full-term and moderately preterm neonates. Detailed subgroup analysis can be visualized in Tables 2-3 and Figure 1.

**Table 3:** Detailed subgroup analysis.

Characteristics	Anal Position Index	P-values
	Mean ± standard deviation	
Gender		
Male	0.36±0.08	<0.001
Female	0.25±0.07	
Birth weight in kg		
≥2.5	0.29±0.09	0.028
<2.5 to ≥1.5	0.34±0.09	
≤1.5 to ≥1.0	0.32±0.10	
<1 kg	0.33±0.08	
Gestational age in weeks		
≥37 to <42weeks	0.29±0.09	0.002
>32 to <37	0.34±0.09	
≥28 to ≤32	0.34±0.1	
<28	0.28±0.05	
Consanguinity		
Yes	0.30±0.10	0.857
No	0.31±0.09	
Maternal co-morbidities		
None	0.30±0.09	0.048
Gestational diabetes	0.32±0.1	
Pregnancy-induced hypertension	0.30±0.08	
Preeclampsia	0.54±0.0	
Hypothyroidism	0.28±0.11	
Hyperthyroidism	0.36±0.0	
PIH and GDM	0.39±0.11	

## Discussion

The API remains a valuable indicator in diagnosing ADA quantitatively, even though the diagnosis remains clinical, viz., inspection at large. Since an individual’s inspection skills are subjective to their experience and largely vary among different ethnicities, an organized manner of diagnosing ADA, as suggested by Reisner et al., remains the yardstick in pediatric surgery. Figure 1 shows the method of measuring the anal position index in girls and boys.

Our study concluded that the API of normal infants was 0.30 ± 0.09 (boys: 0.36 ± 0.07, girls: 0.24 ± 0.06). Any neonate with an API below these indicated values can satisfactorily be diagnosed with having an anterior ectopic anus. According to Rerksuppaphol et al., the risk factors for the development of ADA include female gender, older maternal age, and a later birth order. The higher prevalence of ADA in the female gender is in contrast to the greater prevalence of anorectal abnormalities and Hirschsprung disease in males [9-11].

**Figure 2:**
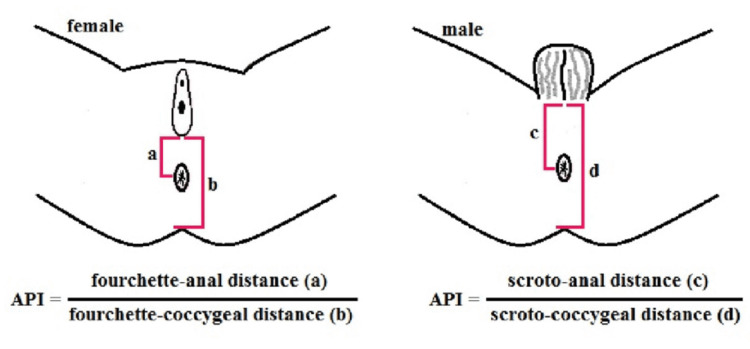
Measurement of anal position index

**Figure 3:**
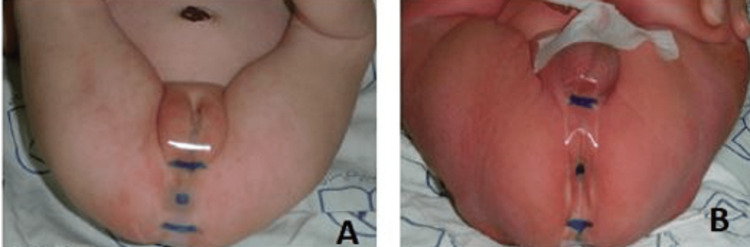
Measuring the inferior border of the coccyx, the middle of the anus, and the fourchette/the first fold of the scrotum.

The presence of ADA has been attributed to chronic constipation refractory to medical management in neonates. The presence of constipation is related to a myriad of causes, mostly functional without having any underlying pathological condition; however, anorectal abnormalities can be a cause of constipation in the early neonatal period and infancy [12,13]. Ishitani et al., in their study of 13 individuals (12 females), reported all children having chronic constipation, which began in the first three to six months of life. Even though the anus had a normal appearance on examination, all patients had marked straining on defecation with concomitant perineal pain. The API of the entire cohort revealed all individuals had ADA and underwent posterior anoplasty with the advancement of the posterior rectal wall, resulting in complete resolution of symptoms [14]. However, this observation remains a bone of contention among scientists globally. Herek et al. commented in their study that constipation was significantly disconnected from having a low API, and the incidence remained similar in individuals with a normal API. The authors hence suggested that ADA is an anatomical variant of the anorectal formation and is not associated with functional outcomes, such as constipation in neonates [4].

Many authors have evaluated the normal position of the anal opening in children in their respective regions. Davari and Hosseinpour [15] performed a cross-sectional study, including 400 individuals, to calculate the API in the Iranian population using the formula suggested by Reisner et al. [6]. The mean API in girls was calculated at 0.45 ± 0.08 (MOU1) (95% CI: 0.44-0.46), and in boys, the score was 0.54 ± 0.07 (MOU2) (95% CI: 0.53-0.55). A mean difference with a standard deviation of 2 was considered to be the normal range for the position of the anus [16]. A detailed review of selected studies evaluating API can be visualized in Table 4.

**Table 4:** A detailed review of selected studies evaluating API.

Reisner et al. [6]	1984	Israel	Newborn +	200 (100 M, 100 F)	0.58 (0.06)	0.44 (0.05)
			4-18 months	30 (15 M, 15 F)	0.56 (0.4)	0.40 (0.06)
Bar-Maor and Eitan [14]	1987	Israel	3 days to 12 years	104 (74 M, 30 F)	0.560 (0.20)	0.39 (0.09)
Genc [12]	2002	Turkey	Newborn	60 (26 M, 34 F)	0.53 (0.05)	0.46 (0.08)
Mohta and Goel [8]	2004	India	Newborn	387 (300 M, 87 F)	0.43 (0.05)	0.37 (0.06)
Davari and Hosseinpour [15]	2006	Iran	Newborn	400 (200 M, 200 F)	0.54 (0.07)	0.42 (0.08)
Rerksuppaphol et al. [10]	2008	Thailand	Newborn	403 (203 M, 200 F)	0.51 (0.07)	0.38 (0.08)
Chan et al. [17]	2009	Taiwan	Newborn	200 (100 M, 100 F)	0.54 (0.03)	0.40 (0.05)
Abdel Salam and Shahin [18]	2011	Egypt	Newborn	400 (200 M, 200 F)	0.48 (0.06)	0.34 (0.08)

With advancements in surgery, a newer tool is being used to diagnose and guide the management of ADA, involving ultrasound imaging. A urethra-to-anus distance (UAD) on perineal ultrasound is being rapidly used as a modality to diagnose anatomical malformations. Bruzeau et al. calculated this distance in females with ADA and less than three years of age to calculate the UAD cut-off points [12]. The UAD calculated for normal infants was 22.9 ± 1.7 mm, and for those with non-operated ADA, it was 21.4 ± 2.4 mm. Individuals with operable ADA had a UAD of 17.5 ± 1.8 mm (a statistically significant difference of p = 0.0001) [16].

## Conclusions

In conclusion, our research sheds light on the crucial role of the API in diagnosing ADA in neonates. ADA, a common congenital abnormality of the anorectal region, is often associated with constipation and can have significant implications for a child's well-being if left untreated. Our findings underscore the significance of the API as a quantitative tool for assessing the position of the anal opening in neonates. By utilizing the API, we have established normative values for Pakistani neonates, with a mean API of 0.30 ± 0.09 for the entire cohort. Notably, we observed a gender-specific difference, with male neonates having a higher mean API of 0.36 ± 0.07 compared to female neonates with a mean API of 0.24 ± 0.06. This gender difference in API highlights potential anatomical variations that may contribute to the diagnosis and management of ADA. Furthermore, our study identifies significant correlations between API and various neonatal factors. The API demonstrated a positive association with gestational age, birth weight, and paternal age. These correlations offer valuable insights into how neonatal development and genetic influences may impact the positioning of the anal opening.

Importantly, the API holds great promise as a practical tool for the early detection and management of ADA. Our results suggest that neonates with an API below the established normative values are at risk of having an anterior ectopic anus. Identifying such cases early on enables timely intervention and appropriate management strategies, potentially preventing long-term functional symptoms such as constipation. As medical practices continue to evolve, our study also highlights the emerging role of ultrasound imaging, specifically the UAD, in diagnosing ADA. This innovative approach provides a cost-effective and accessible means of assessing anal position at the bedside, particularly relevant for resource-constrained settings and low- and middle-income countries. Incorporating ultrasound imaging into the diagnostic process can contribute to more accurate and efficient evaluations, guide clinical decisions, and optimize patient outcomes. In summary, our research contributes to the growing body of knowledge surrounding ADA and its diagnosis in neonates. The API serves as a valuable tool for objectively assessing anal position, with potential applications for patient selection, surgical evaluation, and early intervention. By harnessing the power of quantitative measurements and innovative imaging techniques, we aim to improve the quality of care for neonates with ADA, ultimately ensuring a healthier start in life for these vulnerable individuals.
